# Explant origin and growth regulators as determinants of callus performance and terpenoid indole alkaloid accumulation in *Catharanthus roseus*

**DOI:** 10.1186/s12870-026-08578-8

**Published:** 2026-04-02

**Authors:** Abeer F. Desouky, Salah El-Din El-Assal, Ahmed Abdelsamad, Abdel-Salam Reda, Nancy Danial, Moemen S. Hanafy

**Affiliations:** 1 Plant Biotechnology Department, Biotechnology Research Institute, National Research Center (NRC), Cairo, 12622 Egypt; 2 Department of Genetics, Faculty of Agriculture, Cairo University, Cairo, 12311 Egypt

**Keywords:** Catharanthine, Madagascar periwinkle, Tissue culture, Vinblastine, Vincristine

## Abstract

*Catharanthus roseus* (L.) G. Don (Apocynaceae) is a highly important medicinal plant and a valuable source of diverse pharmacologically active compounds particularly anticancer terpenoid indole alkaloids (TIAs), such as catharanthine (CAT), vinblastine (VBL) and vincristine (VCR). This study investigated the impact of various plant growth regulator (PGR) combinations on callus induction and production of CAT, VBL and VCR. Leaf and stem explants were cultured on media containing seven combinations of auxins and cytokinins, and evaluated for callus induction frequency, biomass accumulation, and alkaloid content after eight weeks. Stem explants exhibited superior responsiveness, achieving 100% callus induction under most PGR treatments, whereas the combination of GA₃ and BAP failed to induce callogenesis in both explant types. HPLC analysis revealed that the combination of 1.5 mg/L 2,4-D and 1.5 mg/L kinetin (Kin) in leaf-derived callus yielded the highest CAT and VBL concentrations (5.01 µg/gFW and 1.96 µg/gFW; respectively). However, VCR notable enhanced in the stem- derived callus grown on a medium containing a combination of 1.0 mg/L each of BAP and NAA (4.71 µg/gFW) and leaf-derived callus grown in a medium containing 1.5 mg/L each of BAP and 2,4-D (2.79 µg/gFW) as compared with the controls (leaves and stems from in vivo grown plants, 1.51 µg/gFW and 0.38 µg/gFW; respectively). Gene expression analysis revealed that the combined application of 1.0 mg/L BAP and 1.0 mg/L NAA enhanced the transcript levels of peroxidase 1 *(PRX*1), secologanin synthase (*SLS*), and strictosidine synthase (*STR*) in stem-derived callus. The upregulation of these genes was positively correlated with increased VCR accumulation. In contrast, the expression levels of octadecanoid-derivative Responsive Catharanthus AP2-domain protein 3 (*ORCA3*) and desacetoxyvindoline 4-hydroxylase (*D4H*) were reduced. These findings highlight the importance of tailored hormonal treatments for optimizing in vitro biomass production and secondary metabolite synthesis in *C. roseus*, offering insights into sustainable alkaloid production for pharmaceutical applications. This study addresses the link between tailored plant growth regulator regimes and the coordinated activation of late-pathway genes, resulting in the selective enhancement of individual TIAs in *C. roseus* callus cultures, thereby providing a novel molecular–biotechnological strategy for targeted alkaloid production.

## Introduction

 Catharanthus is a perennial medicinal plant native to tropical regions and belongs to the family Apocynaceae. The genus comprises eight species, seven endemic to Madagascar (*C. coriaceus*,* C. lanceus*,* C. longifolius*,* C. ovalis*,* C. roseus*,* C. scitulus*, and *C. trichophyllus*) and one species, *C. pusillus*, native to India. Among them, *C. roseus* is the most economically pharmacologically important species and is widely cultivated as both an ornamental and medicinal plant [1]. *C. roseus* serves as a natural chemical factory, synthesizing approximately 200 alkaloids, predominately terpenoid indole alkaloids (TIAs) [2]. Of these, the anticancer alkaloids vinblastine (VBL) and vincristine (VCR) are of exceptional clinical importance and are widely used in the treatment of leukemia, lymphomas, breast cancer, and other malignancies [3–5]. These alkaloids are formed through the coupling of monomeric alkaloids catharanthine (CAT) and vindoline. However, their natural abundance in this plant is extremely low, making their extraction from field-grown plants both costly and economically unfeasible. This limitation arises from spatial separation of the biosynthetic sites and the high degree of cellular specialization in certain leaf cells, where specific steps of the TIAs biosynthetic pathway take place [6, 7]. Besides their anticancer activity, VCR and VBL also exhibit potent antimicrobial activity [8–11].

Due to the very low natural abundance and high commercial value of TIAs, extensive research efforts have focused on alternative production strategies using plant biotechnology tools, allowing precise manipulation of culture conditions to enhance targeted compound synthesis [12]. This approach offers several advantages over conventional field cultivation and chemical synthesis [13]. Various factors have been investigated to improve TIAs production in *C. roseus*, including optimization of in vitro culture media, plant growth regulators, pH, light, carbon sources, temperature, elicitors, precursor feeding, genetic transformation, hairy root cultures, and biotransformation systems [14–19]. Similar strategies have been applied to improve alkaloid accumulation in other medicinal plants such as *Hyoscyamus muticus* [20]. Plant growth regulators (PGRs) play a central role in regulating both callus growth and TIA biosynthesis by influencing key metabolic genes. Cytokinins such as zeatin have been shown to significantly enhance indole alkaloid accumulation in *C. roseus* cell cultures, with responses depending on auxin presence and physiological state [21]. Conversely, auxins can repress key biosynthetic genes such as tryptophan decarboxylase *(TDC)* and strictosidine synthase *(STR*) genes involved in indole alkaloid biosynthesis in *C. roseus* [22, 23]. Several studies confirmed that specific auxin-cytokinin combinations enhance callus growth and increase VBL and VCR production [24, 25].

Recent studies have shown that haploid callus cultures of *C. roseus* derived from anthers was successfully established [26]. Phytochemical profiling confirmed the synthesis of several valuable alkaloids, including ajmalicine, vindolinine, pleiocarpamine, and pericyclivine, demonstrating the potential of haploid cultures as efficient biotechnological platforms for alkaloid production. Likewise, exogenous application of auxins such as indoleacetic acid (IAA), indolebutyric acid (IBA), and naphthaleneacetic acid (NAA) has been shown to enhance alkaloid biosynthesis in several species. For example, in *Annona emarginata*, auxin treatments not only increased total alkaloid content but also altered the alkaloid profile, leading to the discovery of previously unreported compounds [27]. Therefore, the selection of appropriate plant growth regulators (PGRs) is a prerequisite for optimizing callus growth during mass production while enhancing the expression of genes responsible for the biosynthesis of terpenoid indole alkaloids (TIAs).

The production of TIAs in *C. roseus*, including VBL and VCR, is regulated by a complex gene network [28]. Upstream genes in the indole pathway such as Aspidosperma (*AS*) and *TDC* and terpenoid pathway (e.g. secologanin synthase, *SLS*) regulate precursor supply, while downstream genes such as (strictosidine synthase, *STR*, deacetylvindoline 4-O-acetyltransferase, *DAT*, and *PRX*) catalyze the formation of the final alkaloids. Environmental stressors like drought and chemical elicitors such as tryptophan can significantly upregulate these genes, leading to enhanced alkaloid accumulation [29]. Genetic studies showed that *ORCA3* overexpression, either alone or with the enzyme geraniol 10-hydroxylase (G10H**)** in *C. roseus* stimulated the accumulation of monomeric alkaloids such as vindoline and CAT but had limited impact on VBL and VCR accumulation, indicating that late stage dimerization remains a bottleneck requiring further regulation [30]. Likewise, desacetoxyvindoline 4-Hydroxylase (D4H) gene is essential for vindoline biosynthesis but alone cannot raise the final VBL/VCR levels, highlighting the need for coordinated pathway regulation [30]. Despite numerous reports on improving TIA accumulation in *C. roseus* cultures, there is still no consensus on the optimal PGR combinations that simultaneously maximize callus biomass [14], regulate key biosynthetic genes, and enhance individual TIA production. Most studies focus on either growth or metabolite yield but rarely integrate both at the molecular and phytochemical levels. As a result, scalable and efficient in vitro systems for industrial TIA production remain limited. This study addresses this gap by systematically evaluating the effects of selected PGRs on callus growth, TIA biosynthesis (i.e. CAT, VCR and VBL), and the expression of key TIA pathway genes in *C. roseus*, with the aim of developing an optimized in vitro production platform. It is hypothesized that optimized combinations of auxins and cytokinins will significantly enhance callus biomass and modulate key TIA biosynthetic genes, resulting in increased production of VCR and VBL in *C. roseus in vitro* cultures.

## Materials and methods

### Seeds, chemicals, and reagents

Seeds were sourced from the local market. Murashige and Skoog’s (MS) medium [31], 6-benzylaminopurine (BAP), gibberellic acid (GA_3_), kinetien (Kin), indole-3-acetic acid (IAA), 1-naphthaleneacetic acid (NAA), 2,4-dichlorophenoxyacetic acid (2,4-D) and Gelrite were purchased from Duchefa Biochemie (Netherlands). As for authentic standards, catharanthine (CAT) was purchased from Sigma-Aldrich (USA), vinblastine (VBL) was obtained as vinblastine sulfate from United Biotech Pvt. Ltd. (UNIBLASTIN, India), and vincristine (VCR) was obtained as vincristine sulfate from Hikma Pharmaceuticals (Vinracine^®^, London, United Kingdom).

### Seedlings growth condition and treatment

Seeds of *C. roseus* were immersed in 70% ethanol for 30 s. Thereafter, the seeds were surface sterilized for 15 min in commercial Clorox solution (5.4% sodium hypochlorite). The seeds were then rinsed several times with sterile distilled water and germinated on MS medium supplemented with 3% (w/v) sucrose and 0.2% (w/v) gellite, adjusted to pH 5.7. Germination was carried out in a growth chamber maintained at 22 ± 2 °C under cool white fluorescent light with a 16-h photoperiod (50 ± 5 µmol m^− 2^ s^− 1^). For callus induction, leaf and stem explants were excised from four-week-old in vitro-grown seedlings and cultured on solid MS medium containing 30 g/l sucrose supplemented with seven selected combinations of plant growth regulators based on previously published studies on *C. roseus* tissue culture [25, 32–35]. Leaf explants were prepared from fully expanded leaves collected from these seedlings; all selected leaves were morphologically similar to minimize developmental variability. Stem explants consisted of stem segments containing a single internode. The PGR treatments were designed to systematically evaluate the effects of different auxin–cytokinin ratios and hormone types on callus induction and alkaloid biosynthesis. The combinations included balanced, auxin-dominant, and cytokinin-enriched conditions using varying concentrations of BAP, Kin, 2,4-D, NAA, and IAA. Both synthetic auxins (2,4-D and NAA) and the natural auxin (IAA) were included to compare their regulatory effects on biomass accumulation and secondary metabolism, while two cytokinins (BAP and Kin) were used to assess potential differences in cytokinin-mediated responses. In addition, a GA₃ + BAP treatment was incorporated to examine the possible influence of gibberellin on callus growth and terpenoid indole alkaloid production. This experimental design enabled systematic evaluation of hormonal interactions affecting growth and metabolic outcomes. The combinations tested were: 1.5 mg/L 2,4-D + 1.5 mg/L kin; 3.0 mg/L BAP + 2.0 mg/L NAA; 1.0 mg/L BAP + 1.0 mg/L NAA; 0.5 mg/L BAP + 2.0 mg/L 2,4-D; 1.5 mg/L BAP + 1.5 mg/L 2,4-D; 1.5 mg/L kin + 1.5 mg/L IAA and 1.5 mg/L GA₃ + 1.5 mg/L BAP. 

The pH of the media was adjusted to 5.7 prior to sterilization by autoclaving at 121 °C for 20 min. All growth regulators were added after autoclaving. After three weeks of culture, the induced calli were excised from explants, and their induction efficiency was evaluated based on callus induction frequency, morphological characteristics, and fresh and dry weights. The obtained calli (0.5 gFW/Perti dish) were subcultured on the same medium for an additional five weeks to promote proliferation. After eight weeks from the start of culture, callus biomass (fresh and dry weight) and the concentrations of CAT, VBL, and VCR were quantified using high-performance liquid chromatography (HPLC).

### Biomass measurements

Calli were dried by blotting all the external moisture and weighed to determine the final fresh weight (FW). The callus dry weights were also weighed after the callus drying in an oven at 60 °C for 24 h until reaching constant weight.

### Alkaloid extraction and HPLC analysis

Alkaloids were extracted according to the method described by [36]. Briefly, 0.5 g of fresh callus of each sample was transferred into a 10 ml centrifuge tube and soaked in 5 ml of extraction solvent consisting of 2% formic acid in water and methanol (50:50, v/v) for 2 h. The mixture was then sonicated for 30 min and centrifuged at 4000 rpm for 10 min. The resulting supernatant was carefully transferred to a clean tube and filtered through a 0.2 μm PTFE syringe filter before injection into the HPLC system for alkaloid quantification. The alkaloid analysis was performed on an Agilent HPLC system (1260 series) equipped with quaternary pump. The separated was done using zorbax Eclipse plus C18 (150 mm x 4.6 mm), (Agilent, USA), operated at 30 °C. The separation is achieved using a ternary linear isocratic elution with a mobile phase profile of 2% formic acidic: methanol (80:20v/v) with a flow rate of 1.0 ml per min. The injected volume was 20 µl. Variable wavelength detector (VWD) was set at 254 nm and employed for quantification of the alkaloids [37]. Authentic standards of CAT, VCR and VBL were prepared in methanol and used for the preparation of the calibration graphs. Alkaloids were identified by comparing their retention times with those of authentic standards. Quantification was repeated three times for each sample.

### RNA extraction and cDNA preparation

Total RNA was extracted from leaf tissues from wild-type (WT) plant seedlings and callus culture derived from stem on solid medium contain (1.0 mg/L BAP and 1.0 mg/L NAA) (M4S) using TransZol Up kit (TransGen Biotech, Beijing, China Cat. No. ETl 11) according to the manufacturer instructions. To eliminate remaining genomic DNA, the extracted RNA samples were treated with RNase-Free DNase I (Fermentas). RNA was quantified using a nanophotometer (IMPLEN GmbH, Munich, Germany). The OD260/OD280 ratio ranged from 1.8 to 2.0. Then using PrimeScript™ RT Reagent Kit (Takara^®^, Japan) cDNA was synthesized from total RNA (6 µg) in a final reaction volume of 20 µl.

### Quantitative real-time PCR (qRT-PCR) analysis

Quantitative real-time PCR (qRT-PCR) was performed to measure the expression of PRX1 which encoding a class III peroxidase enzyme (PRX1), SLS, STR, D4H and ORCA3 genes involved in TIAs biosynthesis in the stem-derived callus on a media supplemented with a combination of 1.0 mg/L BAP and 1.0 mg/L NAA (M4S), which gives the highest accumulation of VCR. Each 20 µl reaction contained 2 µl of cDNA (40 ng/µl), 1 µl of each forward and reverse PCR primer (10 µM), 5 µl of CAPITAL^™^ qPCR Green Mix, 4x and 11 µl of sterile water. The PCR conditions were set as follows: an initial denaturation at 95 °C for 3 s, followed by 40 cycles of 95 °C for 15s, 60 °C for 30s. The indigenous reference gene 40 S ribosomal protein S9 (*RSP9*) was used as an internal control, and the 2 − ΔΔCt method was applied for relative quantification of gene expression. The primers used in qRT-PCR according to [38] were as follow:

- RSP9: 5’-GAGGGCCAAAACAAACTTGA-3’ and 5’-GAGGGCCAAAACAAACTTGA-‘3.
- PRX1: 5’-CTGCTGCTTCTTCTCATTTCC-3’ and 5’-CAACATCGTTTTGGAAGACCT-‘3.
- STR: 5’-AAAATTCCCGATACTCCG-3’ and 5’-ACCAATGGGCACTTCCTT-3’.
- SLS: 5’-GTTCCTTCTCACCGGAGTTG-3’ and 5’-CCCATTTGGTCAACATGTCA-3’.
- D4H: 5’-TACCCTGCATGCCCTCAACC − 3’ and 5’-TTGAAGGCCGCCAATTGAT-3’.
- ORCA3: 5’-CGAATTCAATGGCGGAAAGC-3’ and 5’-CCTTATCTCCGCCGCGAACT-

### Statistical analysis

The experiments followed a completely randomized design. Statistical analyses were conducted using Python programming language (Python Software Foundation, https://www.python.org) with specialized statistical libraries. A two-way analysis of variance (ANOVA) was performed using the statsmodels package version 0.13.2 [39] to evaluate the main effects of growth regulator treatments and explant types, as well as their interactive effects on alkaloid production and biomass accumulation. For datasets that meeting parametric assumptions, Tukey’s Honestly Significant Difference (HSD) post-hoc test [40] was applied to identify significant differences between treatment means. The significance of differences in gene expression among experimental variants (leaved derived from in vivo plants, control, and callus tissues) was tested using the nonparametric Kruskal-Wallis ANOVA [41], followed by Dunn’s post-hoc test [42] with Bonferroni correction to control the family-wise error rate while detecting specific pairwise differences. Data management and statistical computations were performed using pandas [43] and SciPy [44]. Data visualization was implemented with matplotlib [45] and enhanced using seaborn [46] for improved graphical representation. All the results were calculated as means ± standard error (SE) based on three independent biological replicates. Principal Component Analysis (PCA) was conducted to assess the relationships between biomass parameters (fresh and dry weights) and alkaloid content across 13 callus treatments derived from leaf and stem explants of *C. roseus.* Data processing and statistical analyses were performed in Python (version 3.x) using the pandas library [43] and NumPy [47]. PCA was implemented using the scikit-learn package [48], and the PCA biplot was visualized with Matplotlib [45].

## Results

### Callus initiation and proliferation

**Fig. 1:**
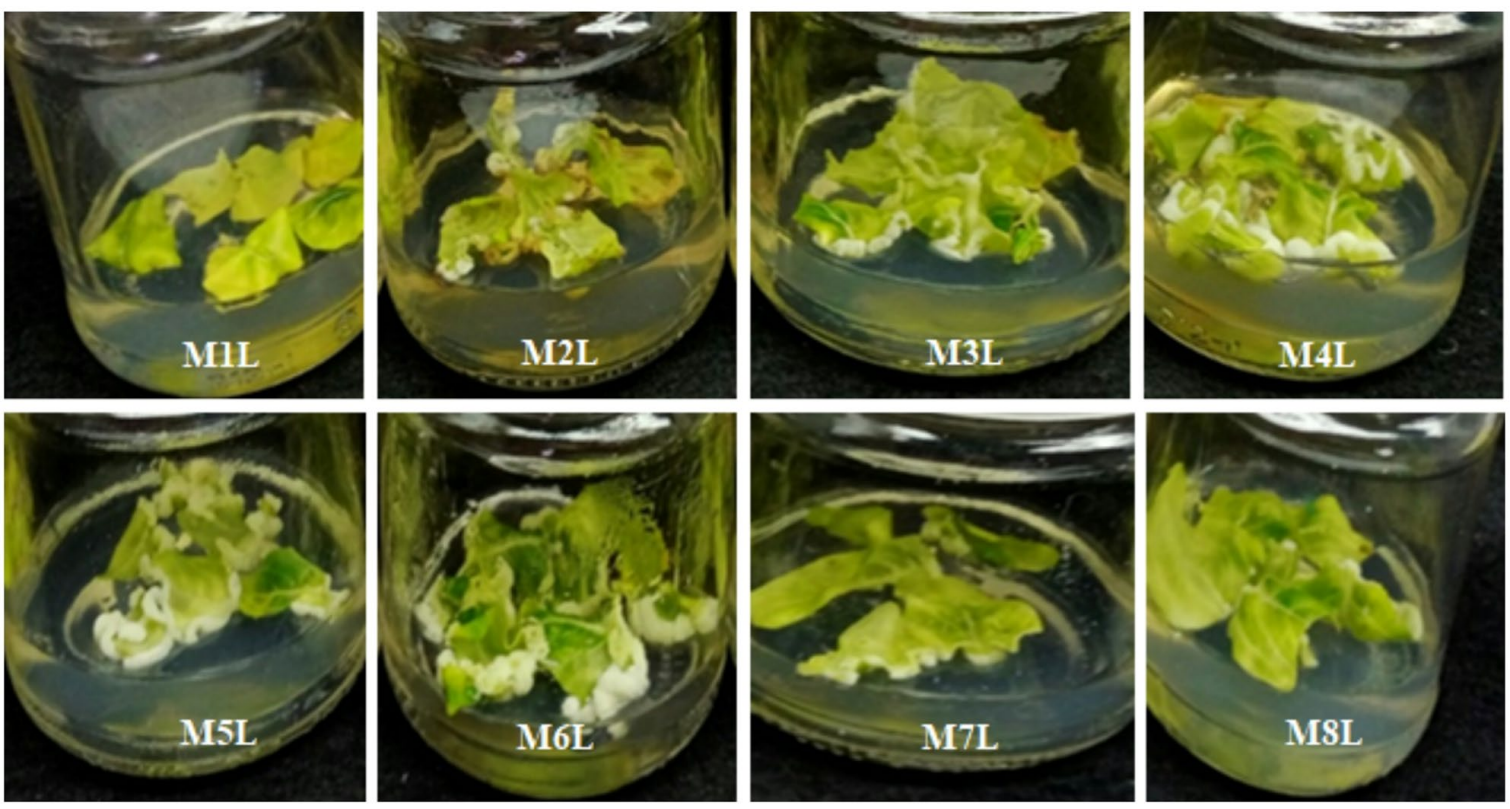
Callus induction from leaf explants of *C. roseus* cultured on Murashige and Skoog (MS) medium supplemented with different plant growth regulator (PGR) combinations after 20 days of cultivation

**Fig. 2:**
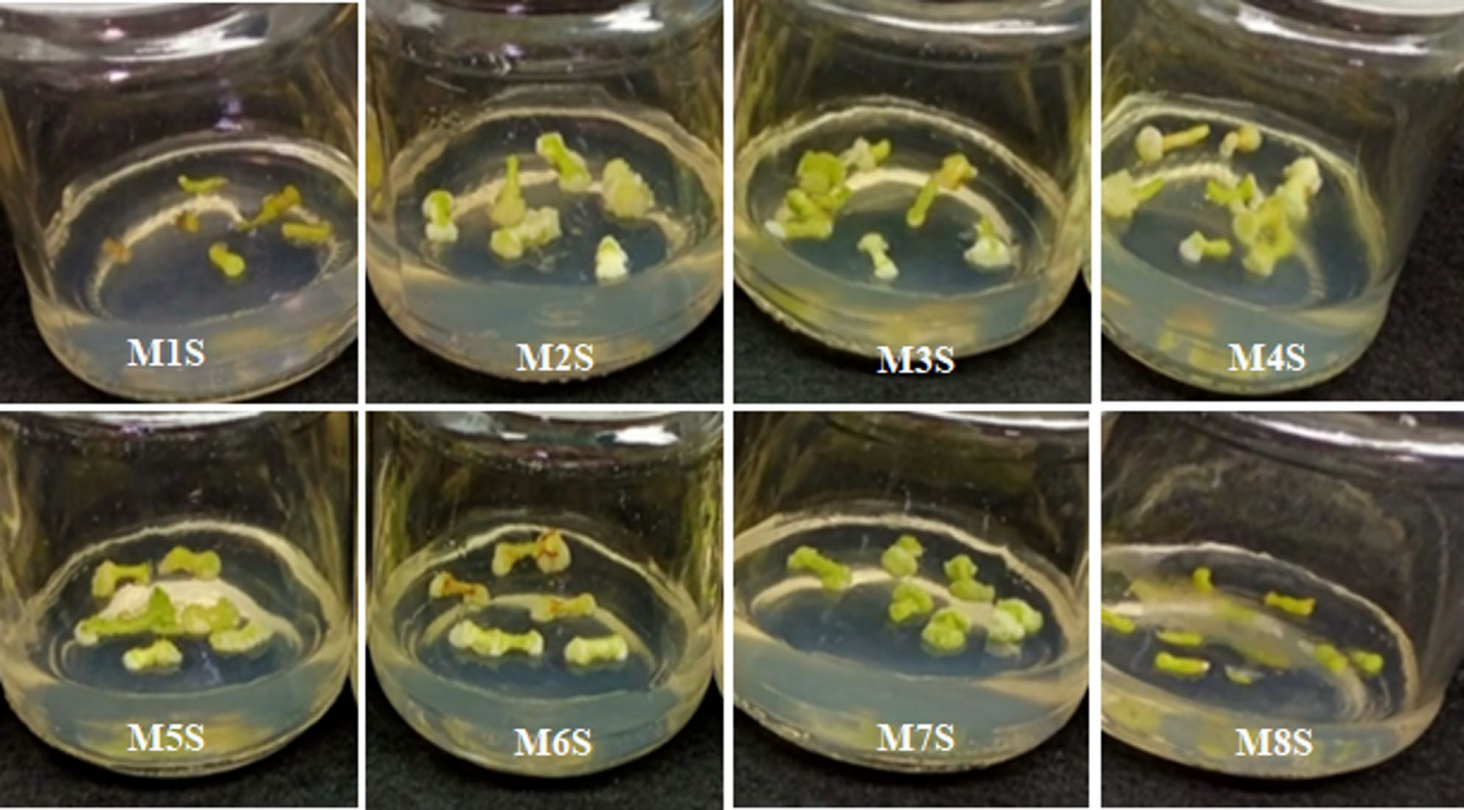
Callus induction from stem explants of *C. roseus* cultured on MS medium supplemented with different PGR combinations after 20 days of cultivation

Stem and leaf explants from in vitro four-week-old *C. roseus* seedlings were used as explants for callus induction. Various combinations of plant growth regulators (PGRs), including 2,4-D, kinetin (Kin), 6-benzylaminopurine (BAP), naphthaleneacetic acid (NAA), indole-3-acetic acid (IAA), and gibberellic acid (GA₃), were tested to optimize callus initiation. The selected PGR types and concentrations were based on previously published protocols reported to promote efficient callus induction. As shown in Table 1, the explant type markedly influenced callus formation under the same media and culture conditions. The control medium, which lacked growth regulators, failed to induce callus formation in either explant type, even after extending the culture period beyond four weeks; eventually, the explants died. In contrast, media supplemented with appropriate PGR combinations significantly enhanced callus induction as well as callus fresh and dry weights (Figs. 1 and 2). However, the combination of 1.5 mg/L GA₃ and 1.5 mg/L BAP did not promote callus induction in either explant type (Table 1). According to our observations, stem explants exhibited a higher callus induction potential than leaf explants under all tested conditions. The combinations of 1.5 mg/L 2,4-D and 1.5 mg/L Kin (M2L) or 3.0 mg/L BAP + 2.0 mg/L NAA (M3L) resulted in 40% and 80% callus induction from leaf explants, respectively, while both combinations produced 100% induction from stem explants (Table 1).

**Fig. 3:**
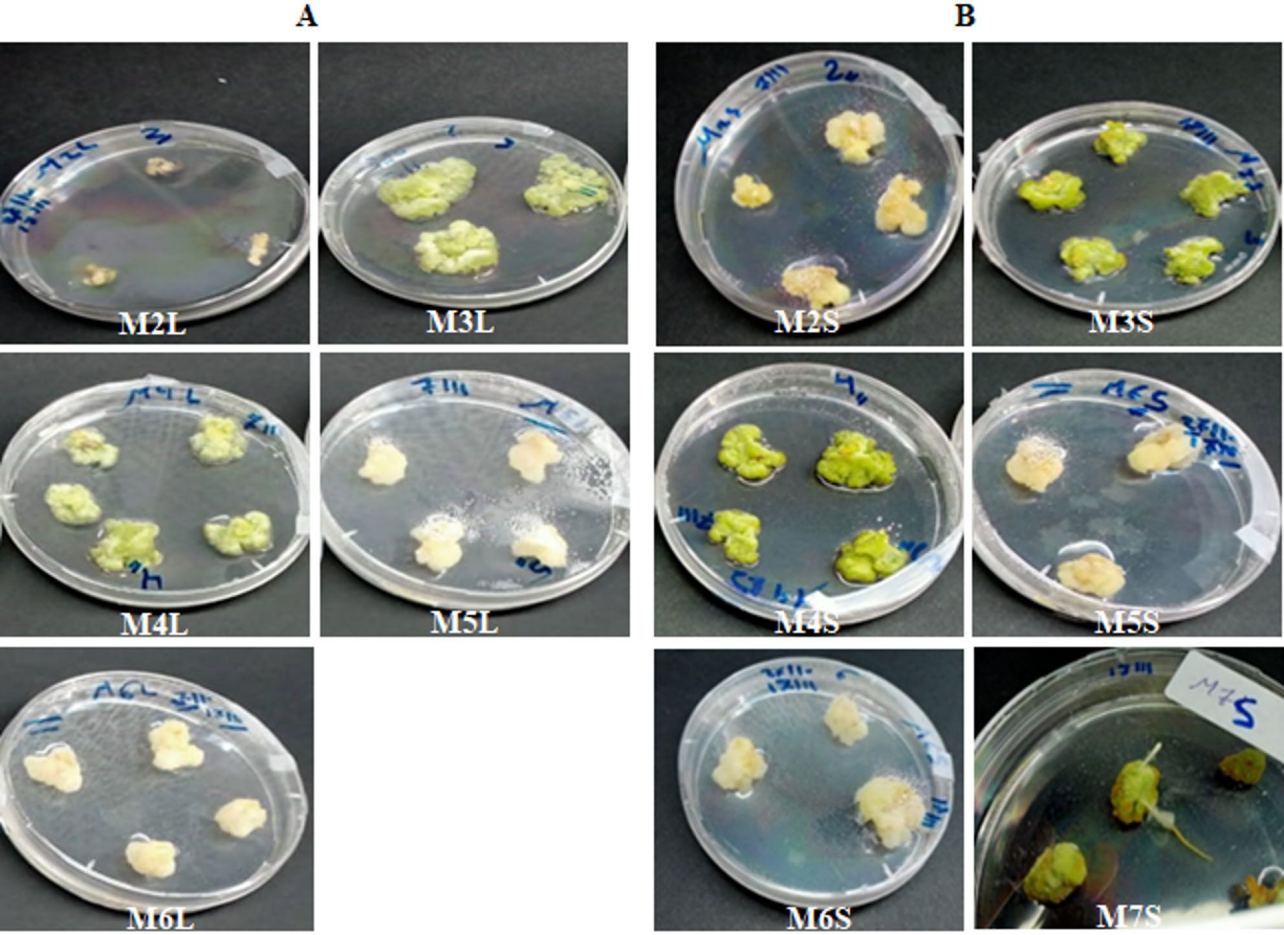
Callus proliferation after 8 weeks of culture on MS medium supplemented with different PGR combinations using leaf (**A**) and stem (**B**) explants

After four weeks of culture, the induced calli differed in texture and color depending on the PGR combination used (Fig. 3; Table 1). Friable creamy calli were observed from both explant types with PGR combinations M2L, M2S, M5L, M5S, M6L, and M6S. In contrast, the remaining combinations produced compact green calli, except for the medium containing 1.5 mg/L each of Kin and IAA (M7L), which failed to induce callus from leaf explants but produced green compact callus from stem tissues (M7S). Considering both induction frequency and callus quality, the highest callus induction rate (100%) in leaf explants was achieved with three media: M4L (1.0 mg/L each of BAP and NAA), M5L (0.5 mg/L BAP + 2.0 mg/L 2,4-D), and M6L (1.5 mg/L each of BAP and 2,4-D). For stem explants, all media combinations induced 100% callus formation except that containing 1.5 mg/L each of GA₃ and BAP. Interestingly, the medium containing the combinations of 1.5 mg/L each of Kin and IAA (M7S) induced root differentiation from stem-derived callus (Fig. 3). However, none of the tested treatments induced adventitious shoot formation from either explant type.

**Table 1:** Effects of various plant growth regulator combinations on callus induction rate from stem and leaf explants of *C. roseus*

MS + supplement	Media code	Type of Explants	Callus induction rate (%)	Callus description (texture and color)
Basal medium (Control)	M1L	Leaf	0	nd
	M1S	Stem	0	nd
1.5 mg/L 2,4-D + 1.5 mg/L kin	M2L	leaf	40	Friable creamy white callus
	M2S	Stem	100	Friable creamy white callus
3.0 mg/L BAP + 2.0 mg/L NAA	M3L	Leaf	80	Compact green callus
	M3S	Stem	100	Compact green callus
1.0 mg/L BAP + 1.0 mg/L NAA	M4L	Leaf	100	Compact green callus
	M4S	Stem	100	Compact green callus
0.5 mg/L BAP + 2.0 mg/L 2, 4-D	M5L	Leaf	100	Friable creamy callus
	M5S	Stem	100	Friable creamy callus
1.5 mg/L BAP + 1.5 mg/L 2,4 D	M6L	Leaf	100	Friable creamy white callus
	M6S	Stem	100	Friable creamy callus
1.5 mg/L kin + 1.5 mg/L IAA	M7L	Leaf	0	nd
	M7S	Stem	100	Compact green callus with roots differentiation
1.5 mg/L GA3 + 1.5 mg/L BAP	M8L	Leaf	0	nd
	M8S	Stem	0	nd

**Fig. 4:**
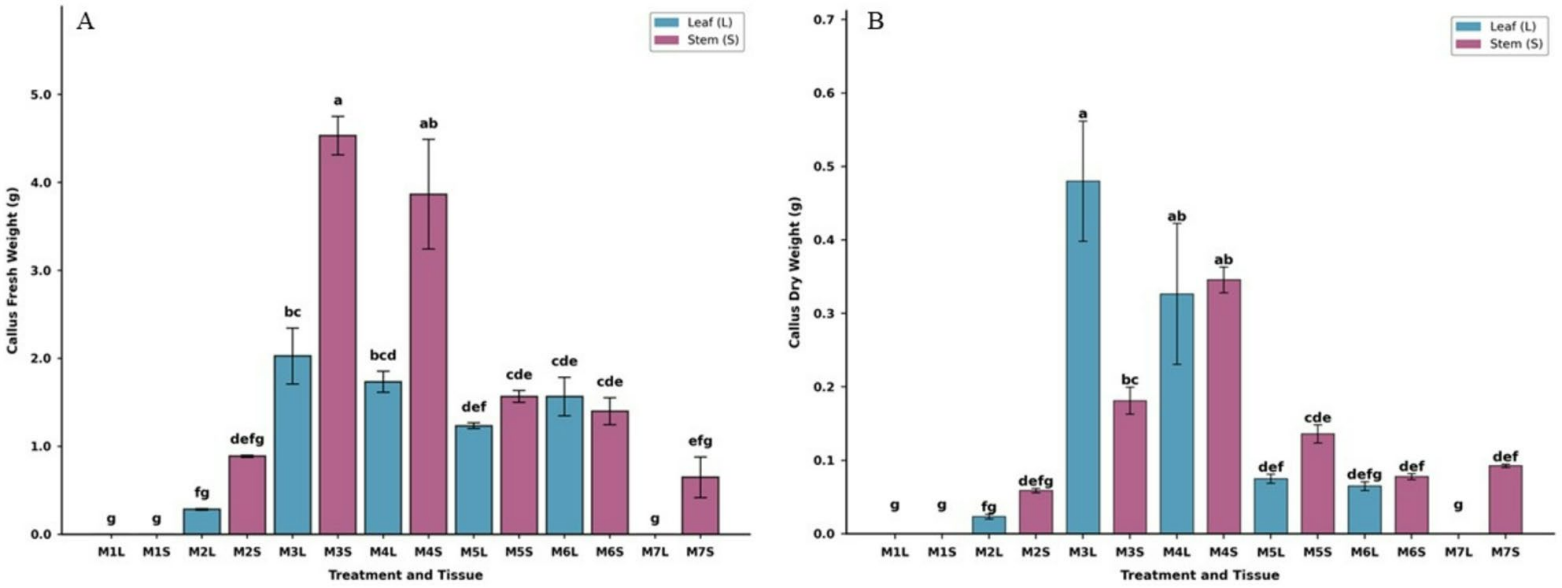
Interaction effects of explant type (leaf and stem) and PGR combinations on callus biomass of *C. roseus* after 8 weeks of cultivation. **A** Fresh weight (FW) and (**B**) dry weight (DW). Values represent mean ± standard error (SE) of three biological replicates (*n* = 3). Data were tested for normality using the Shapiro–Wilk test and for homogeneity of variance using Levene’s test. Two-way analysis of variance (ANOVA) was performed to evaluate the effects of explant type and PGR combinations, followed by Tukey’s honestly significant difference (HSD) test. Different letters indicate statistically significant differences at *p* < 0.05

Figure 4A and B illustrates biomass results (fresh and dry) weights of callus after 8 weeks on solid medium. Figure 4A showed that fresh biomass increases significantly in callus induced from stem explants on media supplemented with PGR combinations especially M3S and M4S reaching 4.5 g and 3.5 g, respectively, in comparison with other PGR combinations and with callus induced from leave explants. While Fig. 4B illustrated the maximum enhancements in the callus dry weights were observed on media supplemented with PGR combinations of M3L, M4L and M4S reaching 0.48 g, 0.33 g and 0.34 g, respectively compared with other growth regulators combinations.

### Effects of plant growth regulators combinations on alkaloids (CAT, VBL and VCR) biosynthesis

Calli of *C. roseus* derived from stem and leaf explants were cultured on media supplemented with various combinations of plant growth regulators, and their effects on the accumulation of CAT, VBL and VCR were investigated. Callus cultures were harvested four weeks after transfer to the fresh media and subsequently analyzed for alkaloid contents. The results presented in Table 2 indicate that the accumulation of these valuable TIAs in *C. roseus* calli was significantly influenced by the applied combinations of plant growth regulators. Regarding CAT, the highest concentration (5.01 µg/gFW) was recorded in calli derived from leaf explants cultured on a medium containing 1.5 mg/L 2,4-D + 1.5 mg/L kin (M2L). Similarly, VBL accumulation reached its maximum levels (1.96 µg/gFW) in calli derived from leaf explants grown on the same PGR combination (M2L), which showed a significant elevation as compared with those observed in in vivo either leaf or stem tissues (controls) (0.562 µg/gFW and 0.45 µg/gFW, respectively) and other tested treatments. Calli derived from stem explants also exhibited enhancement in VBL accumulation, reaching 1.271 µg/gFW in cultures grown on medium supplemented with 1.0 mg/L BAP + 1.0 mg/L NAA (M4S) but without significant difference. For VCR, calli derived from stem explants cultured on a medium containing 1.0 mg/L BAP + 1.0 mg/L NAA (M4S) exhibited significant increase in VCR accumulation (4.714 µg/gFW) compared with leaf or stem tissues derived from in vivo grown plants (1.51 µg/gFW and 0.37 µg/gFW; respectively). Also, callus derived from leaf explants grown on PGR combination of M6L showed a notable accumulation of VCR with a value of 2.79 µg/gFW. However, the other treatments on calli derived from leaf or stem tissues did not show significant differences as observed by ANOVA two ways analysis. Overall, the obtained results emphasizing the importance of optimized hormonal regimes in promoting secondary metabolite biosynthesis in *C. roseus* callus cultures according to the type of explants which produced callus.

**Table 2:** Interaction effects between different plant growth regulator combinations and explant type (leaf- and stem-derived callus) on alkaloid accumulation in *C. roseus* callus

MS + supplement	Media code	Source of callus	Catharanthine (µg/gFW)	Vinblastine (µg/gFW)	Vincristine (µg/gFW)	Callus description (texture and color)
1.5 mg/L 2,4-D + 1.5 mg/L kin	M2L	Leaves	5.01 ± 1.67^a^	1.96 ± 0.30^a^	1.37 ± 0.95^bc^	Friable creamy white callus
	M2S	Stem	1.59 ± 0.15^bcd^	0.52 ± 0.08^bc^	0.33 ± 0.16^c^	Friable creamy white callus
3.0 mg/L BAP + 2.0 mg/L NAA	M3L	Leaves	1.68 ± 0.16^bcd^	1.35 ± 0.76^ab^	1.15 ± 0.45^bc^	Compact green callus
	M3S	Stem	1.99 ± 0.30^bc^	0.49 ± 0.04^bc^	1.51 ± 0.52^bc^	Compact green callus
1.0 mg/L BAP + 1.0 mg/L NAA	M4L	Leaves	1.35 ± 0.12^cde^	0.39 ± 0.02^bc^	0.49 ± 0.02^c^	Friable creamy white callus
	M4S	Stem	1.36 ± 0.24^cde^	1.27 ± 0.87^ab^	4.71 ± 0.05^a^	Friable creamy white callus
0.5 mg/L BAP + 2.0 mg/L 2, 4-D	M5L	Leaves	1.18 ± 0.14^e^	0.44 ± 0.00^bc^	1.21 ± 1.04^bc^	Friable creamy white callus
	M5S	Stem	1.64 ± 0.12^bcd^	0.44 ± 0.03^bc^	2.16 ± 0.09^ab^	Friable creamy white callus
1.5 mg/L BAP + 1.5 mg/L 2,4 D	M6L	Leaves	1.26 ± 0.27^de^	0.46 ± 0.02^bc^	2.79 ± 2.06^a^	Friable creamy white callus
	M6S	Stem	1.24 ± 0.15^de^	0.14 ± 0.12^c^	0.65 ± 0.31^c^	Friable creamy white callus
1.5 mg/L kin + 1.5 mg/L IAA	M7S	Stem	2.13 ± 0.09^b^	0.28 ± 0.15^c^	1.05 ± 0.30^bc^	Compact green callus
In vivo leaves (control)			1.43 ± 0.03^cde^	0.56 ± 0.03^bc^	1.51 ± 0.72^bc^	
In vivo stems (control)			1.56 ± 0.144^bcd^	0.45 ± 0.02^bc^	0.38 ± 0.12^c^	

Values are mean ± SE (standard error) of three replicates. Values with same letters are not significantly different between themselves(*P * 0.05) as determined by Tukey's test

### Principal component analysis (PCA)

**Fig. 5:**
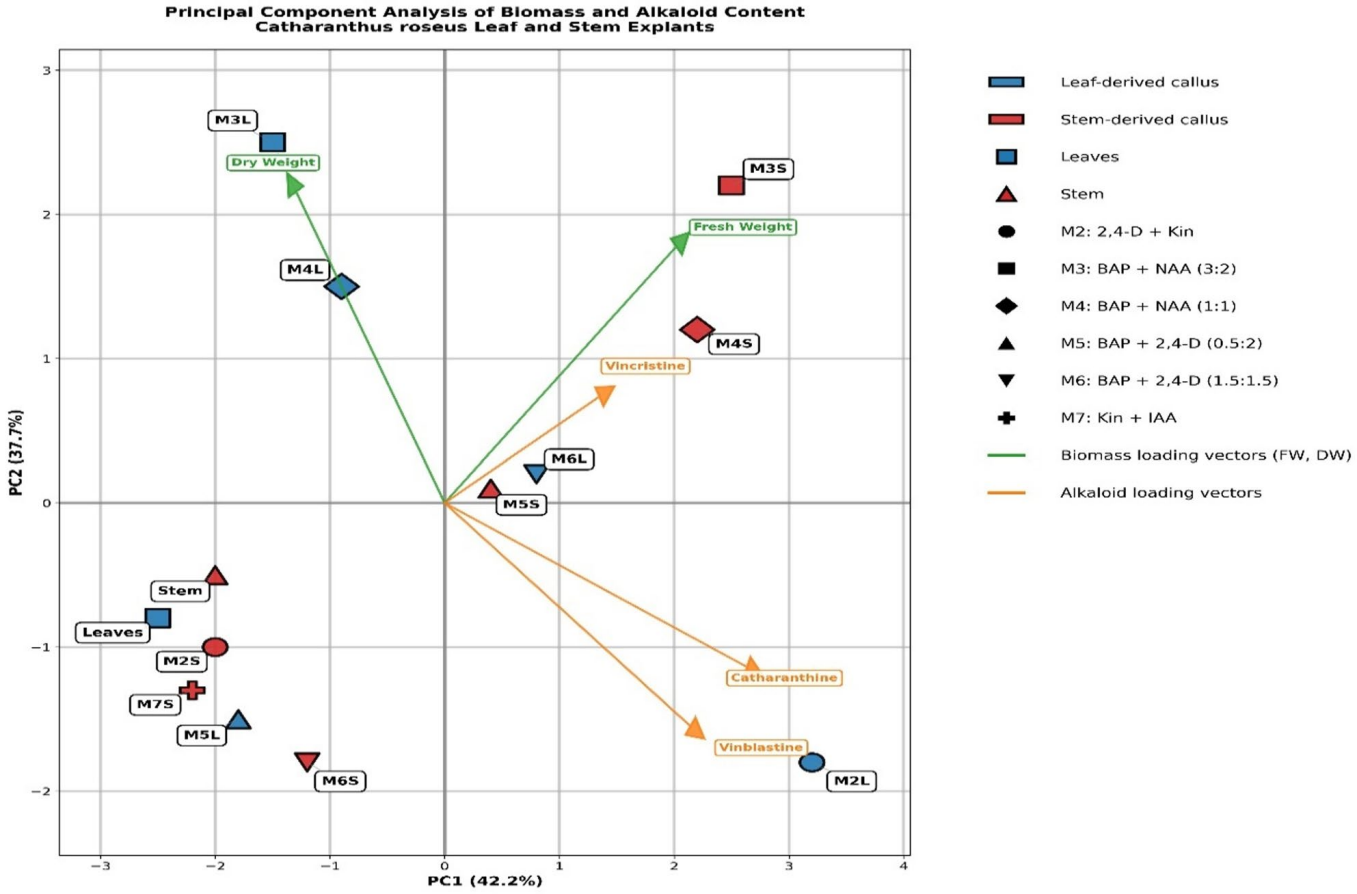
PCA biplot showing the association between biomass parameters and alkaloid content in callus cultures of *C. roseus* derived from leaf (L) and stem (S) explants under different plant growth regulator (PGR) treatments (M1–M7). The first two principal components explained 79.9% of the total variance (PC1 = 42.2%, PC2 = 37.7%). Green vectors represent biomass traits (FW and DW), and orange vectors represent alkaloid content (CAT, VBL, and VCR). Vector length indicates the contribution of each variable to the principal components, and the angle between vectors reflects correlations among variables. Treatments positioned closer to a given vector are positively associated with that variable. L, leaf-derived callus; S, stem-derived callus

PCA was conducted to examine the relationships between biomass accumulation (FW and DW) and alkaloid production (CAT, VBL, and VCR) across 13 callus treatments derived from leaf and stem explants of *C. roseus*. The first two principal components accounted for 79.9% of the total variance, with PC1 explaining 42.2% and PC2 explaining 37.7%. PC1 primarily distinguished treatments based on alkaloid accumulation, exhibiting strong positive loadings for VBL and CAT, with an opposing association with dry weight. PC2 was mainly associated with biomass production, particularly fresh weight, and reflected variation in growth performance among treatments. The biomass loading vectors (FW and DW) were oriented toward the upper region of the biplot, with dry weight closely aligned with M3L, indicating its strong association with enhanced dry biomass production. Alkaloid loading vectors were directed toward the right quadrant, with M2L (2,4-D + Kin 1.5:1.5 on leaf explants) strongly associated with CAT and VBL accumulation, while M4S (BAP + NAA 1:1 on stem explants) was positioned in proximity to the VCR vector, corresponding to its elevated VCR content. Treatments such as M3L, M4L, and M3S clustered in the upper region of the plot, reflecting strong biomass responses, whereas M6L and M5S were located near the origin, indicating intermediate metabolic performance. Treatments positioned in the lower left quadrant, including M5L, M2S, M6S, and M7S, exhibited comparatively lower biomass and alkaloid accumulation. Overall, the PCA demonstrates that specific plant growth regulator/explant combinations drive distinct metabolic profiles in *C. roseus* callus cultures, highlighting differential optimization of biomass production and secondary metabolite biosynthesis fig. 5.

### Quantitative real-time PCR

**Fig. 6:**
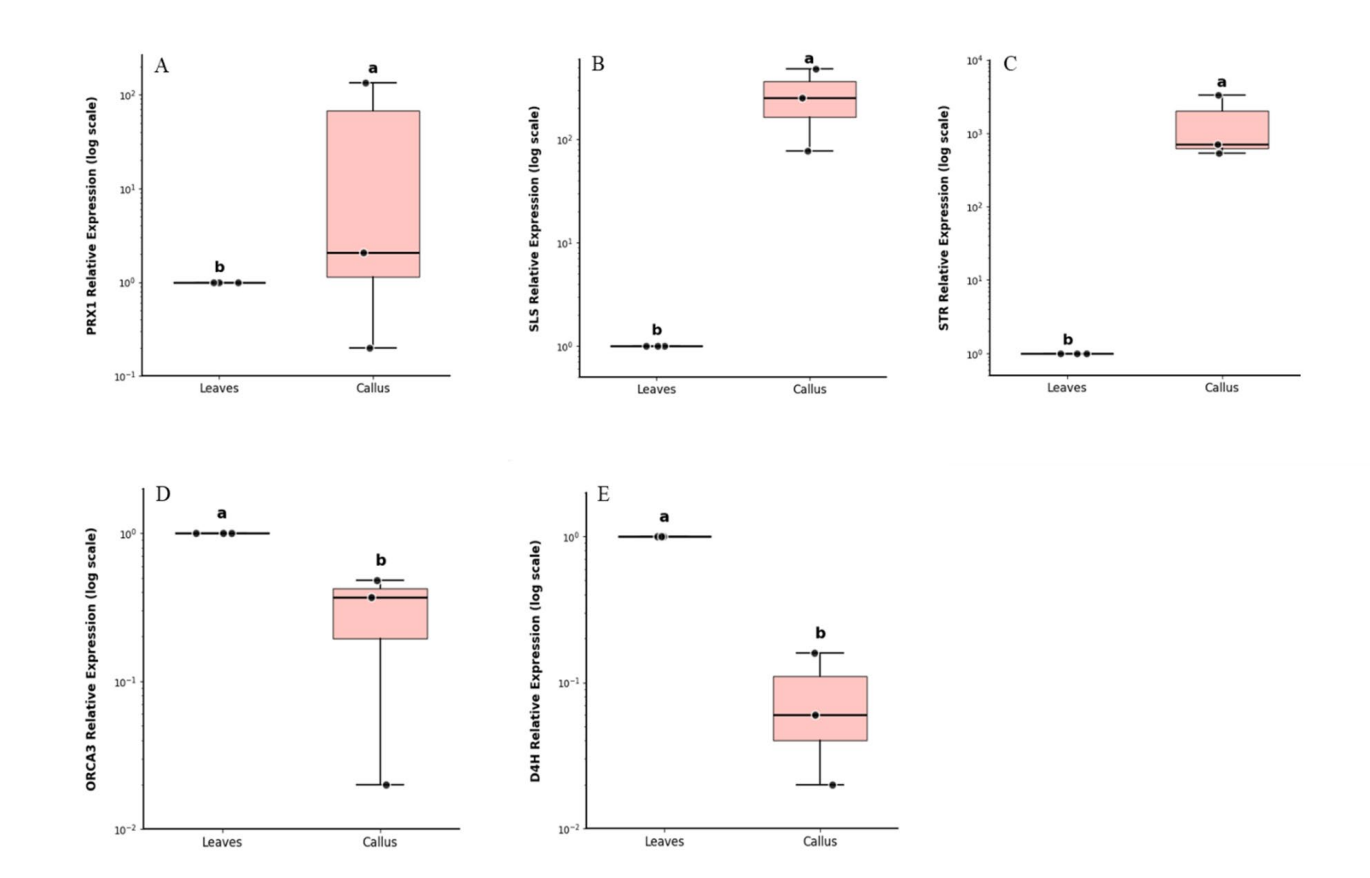
Relative expression levels of key terpenoid indole alkaloid biosynthetic genes in *C. roseus*. **A** PRX1, **B** SLS, **C** STR, **D** ORC3, and **E** D4H relative expression in in vivo-derived leaves (control) and stem-derived callus cultured on M4S medium. Data are presented as box plots showing three biological replicates (*n* = 3). Different letters indicate statistically significant differences at *p* < 0.05

Data presented in Fig. 6 show that using plant growth regulator combinations of 1.0 mg/l BAP and 1.0 mg/L NAA (M4S) resulted in a significant enhancement in the relative expression of alkaloid (CAT, VBL and VCR) key biosynthesis genes (PRX1, SLS and STR) in the stem-derived callus compared with their expression in in vivo leaf tissues (the main source of these important TIAs). This transcriptional upregulation was consistent with the increased accumulation of VBL and VCR observed under the same treatment (Table 2), although the increase in VBL was not statistically significant. Despite the elevated expression of PRX1, SLS, and STR, no corresponding increase in CAT accumulation was detected (Table 2; Fig. 6). Conversely, the transcript levels of *ORCA3* and *D4H* were significantly reduced under M4S treatment (Fig. 6); however, this reduction did not negatively affect alkaloids accumulation (Table 2).

## Discussion

The effects of explant type and specific plant growth regulator combinations on callus induction and proliferation were investigated to identify the most suitable explant for establishing callus cultures and, ultimately, for producing and enhancing the production of valuable medicinal plant compounds from *C. roseus*. The obtained results revealed that stem explants exhibited higher responsiveness and produced significantly greater callus biomass compared with leaf explants. The texture and the color of the callus were depended on the PGR combinations, either friable creamy or compact green callus. It was observed that in the presence of 2, 4-D, both stem- and leaf-derived calli were friable creamy regardless the type of the combined cytokinin. However, in the absence of the 2, 4-D, the produced calli turned to green compact. These results aligned with a well-documented phenomenon, where auxins like 2,4-D typically promote friable, non-organogenic callus, while a balanced or cytokinin-leaning ratio often supports more organized, compact, and sometimes chlorophyllous tissues [13, 49, 50]. Constant with our findings, Elateeq et al. [51] demonstrated that a combination of 1 mg/L NAA and 2 mg/L BA in the callus induction medium of *Ginkgo biloba*, yielded the highest percentage of callogenesis from leaf explants. Moreover, this growth regulators balance promoted the formation of a compact callus exhibiting variable pigmentation under both a 16/8 h photoperiod and continuous darkness, suggesting that the auxin-cytokinin interaction played a decisive role in modulating callus morphology and quality.

Our results showed that the highest callus induction percentage, fresh weight (FW) and dry weight (DW) were observed in stem explants cultured on MS medium supplemented with either 3.0 mg/L BAP + 2.0 mg/L NAA (MS3) or 1.0 mg/L each of BAP and NAA (M4S). However, the combination of 1.5 mg/L each of BAP and GA3 did not produce callus form both explants. Additionally, the combination of 1.5 mg/L each of kinetin (kin) and IAA produced 100% callus from stem explants and promotes root induction from the callus, however, this combination failed to induce callus from leaf explants. Numerous studies have shown that explant type and the hormonal regime strongly determine callus induction efficiency in *C. roseus* [14]. The obtained results agree with Singh et al. [32], who reported a 99% callus induction rate from hypocotyl explants cultured on MS medium supplemented with 1.0 mg/L BAP and 1.0 mg/L NAA. These findings support the established principle that a balance between auxins and cytokinins is essential for dedifferentiation and callus proliferation. In our study, the obtained results revealed that the superior performance of stem explants, achieving up to 100% induction frequency across most of PGR treatments compared to leaf explants, suggests that stem tissues possess a higher inherent morphogenetic potential or a more favorable endogenous hormonal balance for callus initiation. This is strongly supported by the findings of Abbasi et al. [52], who reported that callus induction from stem explants of *Isodon rugosus* was close to 100% with specific PGR combination, while leaf explants showed lower responsiveness. Furthermore, their study confirmed that stem-derived calli also accumulated significantly higher biomass and valuable secondary metabolites. The complete failure of callus induction in leaf explants on media containing either kin + IAA (M7L) or GA3 + BAP (M8L) indicates that these specific combinations are ineffective in triggering the dedifferentiation process in leaf explants. Conversely, combinations of BAP with either NAA or 2,4-D were highly effective for callus induction from leaf explants, which is consistent with previous reports on *C. roseus* and other medicinal plants [16, 53]. Collectively, these findings highlight the importance of selecting a responsive explant type and tailoring the PGR combination to maximize callus induction in *C. roseus* [34, 54, 55].

Although callus induction was an essential preliminary step, the primary objective of this study was to enhance the production of valuable anticancer alkaloids. Two-way ANOVA was applied to evaluate the combined effects of explant type and PGR combinations on TIA accumulation in *C. roseus* callus. HPLC analysis revealed that alkaloid accumulation was frequently independent of biomass growth, underscoring the well-recognized disconnection between primary growth and secondary metabolism in plant cell cultures [13]. The use of specific PGR combinations provided a targeted strategy to selectively enhance the production of individual alkaloids. This selective enhancement, where certain PGR combinations favor specific branches of the terpenoid indole alkaloid pathway, has been demonstrated in previous studies on *C. roseus* [25] and is consistent with the broader role of phytohormones in differentially regulating complex metabolic networks [3]. For instance, the established hormonal regulation of this pathway notes that auxins like 2,4-D can strongly inhibit general biosynthesis, while cytokinins like BAP and other regulators like methyl jasmonate (MeJA) can stimulate and shift the production toward specific compounds [13]. These findings confirm that optimized PGR combinations provide an effective method for directing the biosynthesis of targeted pharmaceutical alkaloids.

The obtained results revealed that the medium M2L (1.5 mg/L 2, 4-D + 1.5 mg/L kin) yielded the highest CAT and VBL accumulation in leaf-derived callus. However, this combination was less effective for VCR. Also, this combination did not promote the accumulation of TIAs in the stem-derived callus. These results in accordance with those reported by Mekky et al. [25], who also reported that a combination of kin and 2,4-D suppressed the production of VCR, suggesting that the 2,4-D-induced pathway that may favor the synthesis of early precursors like CAT and VBL but not the later, more complex dimerization steps leading to VCR. This result is strongly supported by Kalidass et al. [56], who found that *C. roseus* callus cultures can produce substantial amounts of VCR, with yields highly influenced by specific auxin and cytokinin combinations. The obtained results revealed that the combination of BAP and NAA with 1.0 mg/L each proved particularly potent for the dimeric alkaloids. This combination was optimal for VCR accumulation in stem- derived callus but not the case with leaf-derived callus. This synergistic effect of cytokinin and auxin aligns with the findings of Mekky et al. [25], who demonstrated that kin and IAA upregulated the synthesis of these alkaloids. The efficacy of NAA in enhancing alkaloid content is further corroborated by recent studies in other species. For example, Mimi et al. [27] showed that exogenous application of auxins like NAA significantly increased alkaloid accumulation and altered the metabolic profile in *Annona* species, highlighting the broad applicability of auxins in metabolic engineering for secondary metabolites.

The superior VCR production observed in this study, particularly in media containing cytokinins like BAP (e.g., M4S, M6L), can be explained by the hormone’s role in activating key biosynthetic genes. These results are strongly supported by the work of Papon et al. [57], who demonstrated that cytokinins specifically upregulate the terpenoid precursor branch of the TIA pathway in *C. roseus* suspension cells. They found that cytokinin treatment markedly enhanced the expression of geraniol 10-hydroxylase (G10H), a critical cytochrome P450 enzyme, and, in synergy with ethylene, also boosted the expression of genes in the methyl-erythritol phosphate (MEP) pathway (DXS, DXR, MECS). This suggests a direct molecular rationale for the findings reported here which is the inclusion of BAP in the PGR combinations likely stimulated the flux of terpenoid precursors by activating these early pathway genes, thereby overcoming a major bottleneck, and facilitating the increased synthesis of alkaloids like CAT, VBL and VCR that were measured. This confirms that cytokinins are not merely permissive for growth but are active regulators that potently stimulate the metabolic machinery for alkaloid production. The explant source of the callus proved to be a decisive factor for alkaloid production. Stem-derived callus, particularly under M4S, accumulated significantly higher VCR compared to leaf-derived callus. This observed variation, where the callus source dictates the callus’s metabolic potential, can be attributed to the differential expression of biosynthetic pathway genes inherent to the source tissue, a phenomenon well-documented in *C. roseus* [3]. Furthermore, the demonstrated capacity of these undifferentiated callus cultures to produce complex TIAs is highly promising. It confirms that the biosynthetic machinery remains functional in vitro, a finding that resonates with and is supported by the success of other organ-derived callus systems, such as anther-derived haploid callus, in synthesizing a diverse array of bioactive alkaloids [26].

Recent in vitro studies demonstrate that alkaloid accumulation in callus and suspension cultures can be significantly increased by elicitors and optimized culture conditions. For example, fungal elicitation with *Fusarium oxysporum* increased VBL and VCR production in embryogenic tissues of *C. roseus*, with VBL reaching approximately 1.27 µg/g DW and VCR reaching 0.31 µg/g DW. These values represented increases of 7.88% and 15.50%, respectively, compared to non-elicited controls [58]. More recently, a response surface methodology (RSM)-based optimization study evaluated the combined effects of LED light quality, cadmium stress, glycine supplementation, and exposure time in *C. roseus* cell suspension cultures. Under optimized elicitor conditions, VBL accumulation reached 9.41 mg/g DW, while VCR accumulation reached 22.73 µg/g DW [59]. Compared with these elicitor-driven systems, our study demonstrates that targeted plant growth regulator (PGR) combinations without heavy metal or chemical elicitation can achieve substantial alkaloid accumulation. Notably, VCR reached 4.71 µg/g FW under 1.0 mg/L BAP + 1.0 mg/L NAA in stem-derived callus, placing our system within the upper performance range of contemporary in vitro systems in recent literature.

Our results indicated a significant upregulation of the peroxidase 1 (PRX1), secologanin synthase (SLS) and strictosidine synthase (STR) genes in stem-derived callus tissues, as compared with its expression in vivo leaves, under PGR combinations of 1.0 mg/L BAP and 1.0 mg/L NAA offers a mechanistic understanding of the physiological conditions that promote VCR accumulation. These results agree with previous report of Perveen et al. [60], which showed that VCR accumulation was associated with the upregulation of key terpenoid indole alkaloid biosynthetic genes, such as *PRX1*, suggesting that PRX1 coordinated a role in the alkaloid’s production. Also, Canel et al. [61] reported that boosting the expression of crPRX1 correlates with higher VBL levels and reduced hydrogen peroxide (H₂O₂) in callus tissues. This result confirms that upregulating critical enzymatic steps in the alkaloid biosynthetic pathway leads to increased alkaloid accumulation. Also, our findings align with those obtained by Canel et al. [61] and Wang et al. [62], which identified the STR enzyme as a major bottleneck and showed that its over-expression could dramatically increase alkaloid yields. According to earlier research, the SLS gene is a key enzyme in the terpenoid pathway, which provides the terpenoid precursor **(**secologanin**)** for the synthesis of TIAs. Overexpression of the *SLS* gene enhances secologanin availability and consequently stimulates the accumulation of TIAs [29]. This is consistent with our results, where *SLS* overexpression was associated with enhanced accumulation of VCR. The obtained results of the current study highlight a critical disconnect between the expression of specific biosynthetic genes and the final production of dimeric alkaloids. Although octadecanoid-derivative Responsive Catharanthus AP2-domain protein 3 (ORCA3) and desacetoxyvindoline 4-hydroxylase (D4H) are well recognized for their central roles in the terpenoid indole alkaloid pathway and acting as a master transcriptional regulator and a key enzyme in the late steps of vindoline biosynthesis, respectively, their expression levels were significantly reduced. Importantly, this reduction did not negatively affect the biosynthesis of VBL and VCR. suggesting that the regulation of dimeric alkaloid production is not directly dependent on the transcriptional activity of these upstream components. Similar observations have been reported previously, indicating that increasing precursor supply through ORCA3- or D4H-mediated regulation alone is insufficient to enhance dimeric alkaloid yields [30]. These findings suggest that TIA biosynthesis is limited by coordinated pathway bottlenecks rather than transcriptional activation alone, and that balanced regulation of key enzymatic steps is required to effectively increase metabolic flux [63]. These findings support the hypothesis that major metabolic bottlenecks occur downstream of precursor biosynthesis, particularly during the highly compartmentalized dimerization process or the intercellular transport of monomeric intermediates. Consequently, the pathway appears to be buffered at this stage, rendering dimeric alkaloid accumulation relatively insensitive to fluctuations in upstream gene expression. Although ORCA3 is known to regulate D4H expression within the jasmonate-responsive pathway, the present results emphasize that effective enhancement of VBL and VCR production will likely require targeted manipulation of late-stage enzymatic steps and cellular transport mechanisms rather than upstream transcriptional regulators alone.

## Conclusion

The obtained results indicate that there is no single universal PGR formulation for maximizing all aspects of *in vitro C. roseus* culture. Instead, the hormonal regime must be strategically tailored based on the target metabolite. The combination of 2,4-D and kinetin favors CAT and VBL production in leaf-derived callus, whereas balanced combinations of BAP and NAA are more effective for stimulating the biosynthesis of the pharmaceutically critical dimeric alkaloids, VCR in stem-derived callus. This work provides a framework for optimizing in vitro protocols to sustainably produce these high-values, low-abundance alkaloids, thereby supporting the development of plant cell culture-based biotechnological platforms. In future research, comprehensive gene expression and transcriptomic studies should be conducted to elucidate the molecular mechanisms by which specific plant growth regulator (PGR) combinations regulate alkaloid biosynthetic pathways. Furthermore, scaling-up studies should be carried out to maximize the production of terpenoid indole alkaloids (TIAs) employing advanced plant biotechnology tools to meet the demands of the pharmaceutical industry.

## Data Availability

All data generated or analysed during this study are included in this published article.
